# Silencing of *vitellogenin* gene contributes to the promise of controlling red palm weevil, *Rhynchophorus ferrugineus* (Olivier)

**DOI:** 10.1038/s41598-021-01159-9

**Published:** 2021-11-04

**Authors:** Khawaja G. Rasool, Khalid Mehmood, Muhammad Tufail, Mureed Husain, Waleed S. Alwaneen, Abdulrahman S. Aldawood

**Affiliations:** 1 Plant Protection Department, College of Food and Agriculture Sciences, King Saud University, Riyadh, 11451 Saudi Arabia; 2 Ghazi University, Dera Ghazi Khan, Punjab Pakistan; 3 Institute of Plant Protection, Muhammad Nawaz Shareef (MNS) University of Agriculture, Multan, 60000 Punjab Pakistan; 4 National Center for Agricultural Technology (NCAT), King Abdulaziz City for Science and Technology (KACST), Riyadh, Saudi Arabia

**Keywords:** Biotechnology, Molecular biology, Plant sciences

## Abstract

Red palm weevil [*Rhynchophorus ferrugineus* (Olivier)], is native to South Asia and expanding its distribution range globally. Recent invasions of red palm weevil around the world, including Saudi Arabia, has become a global constraint for the production of palm species. Although, several control measures have been tested, none of them seemed successful against this invasive species. Therefore, we focused on silencing the reproduction control gene *vitellogenin (Vg)* based on RNA interference (RNAi) strategy for its possible application in the management of *R. ferrugineus*. The *Vg* is a major yolk protein precursor critical for oogenesis. To do this, fat body transcriptome of *R. ferrugineus* female adults was sequenced, which provided partial *Vg* gene transcript (FPKM 5731.60). A complete *RfVg* gene transcript of 5504 bp encoding 1787 amino acids was then sequenced using RCAE-PCR strategy and characterized. Phylogenetic analysis suggested that *RfVg* has closer ancestry to the coleopteran insects. The *RfVg*-based RNAi significantly suppressed the expressions of *Vg* gene. The 15, 20 and 25 days post-injection periods suppressed *Vg* expressions by 95, 96.6 and 99%, respectively. The suppressed *Vg* expressions resulted in the dramatic failure of *Vg* protein expression, which caused atrophied ovaries or no oogenesis and ultimately eggs were not hatched. These results suggest that knockdown of *Vg* gene involved in *R. ferrugineus* reproduction is a promising target for RNAi-based management of *R. ferrugineus*.

## Introduction

Date palm, [*Phoenix dactylifera* (Linnaeus, 1753)] is one of the oldest fruit trees in the Arabian Peninsula and major economic fruit crop in Saudi Arabia (SA). Date palm is cultivated on an estimated area of 156,848 hectares^1^ in SA with an annual production of 1,050,000 MT^2^. Unfortunately, palm trees are at high risk of red palm weevil [*Rhynchophorus ferrugineus* (Olivier) (Coleoptera: Dryophthoridae)] infestation, which is the most noxious pest of palm trees.

*Rhynchophorus ferrugineus* causes severe damages to palm trees while spending its entire life cycle inside the tree^3^. This species commonly attacks and feeds on young date palm trees, which are less than 20 years of age^4^. Heavy economic losses have been caused by *R. ferrugineus* infestation throughout the world during last few decades^4^. Females of *R. ferrugineus* can lay 270–396 eggs^5^. Generally, eggs are hatched within 3 to 5 days and emerging larvae start boring into the interior of the palms. The advanced damage to the palms results in the mortality of trees^3^. Egg production in *R. ferrugineus* depends on major yolk protein precursor -vitellogenin (Vg)- and its uptake by developing oocytes^6^. Although, several strategies, i.e., chemicals, entomopathogens, and pheromone traps^7–10^ have been used to control *R. ferrugineus*, all of these were unable to control the pest. The hidden nature of the pest is the possible reason of failed control by these management practices. Continuous and non-judicious application of synthetic chemicals against insect pests have posed adverse effects to human health, caused environmental pollution, and resulted in the evolution of resistant populations of several pests^11,12^. Nonetheless, excessive pesticides’ use in the field negatively affect the populations of predators and parasites of harmful insects^11,13^ through non-target effects. Consequently, technologies capable of suppressing insect pest populations in an environment-friendly manner, such as molecular approaches are direly needed for environment-friendly and sustainable management of insect pests. Although *R. ferrugineus* is a crucial pest of date palm, its reproduction mechanism at molecular level remains elusive. The *Vg* gene encodes major yolk protein precursor -*vitellogenin* (*Vg*)- and plays a critical role in effective reproduction of all oviparous organisms, including insects. In insects, the *Vg* is expressed in the female fat body cells^6,14–16^, which is then translated and Vg is secreted into the hemolymph and finally sequestered by oocytes through a particular receptor called Vg receptor (VgR) by endocytosis^17–21^. Once *Vg* enters the oocytes, it is stored in crystalline form as vitellin (Vn) and used as a major protein for developing embryo^22–24^. The *Vg* genes have been characterized at molecular and genetic levels from several insect species, including cockroaches^14,15,25^, giant water bug ^26^ and rice brown planthopper^27^. It is proposed that *Vg* could be an ideal target for future pest control strategies.

Since *Vg* gene plays a critical role in the reproduction and enhance population of insects, disruption of *Vg* gene expression is believed to hold a significant potential as an effective control measure for insects. Therefore, RNA interference (RNAi)-based species-specific insecticide could be convenient and effective. The RNAi is a versatile technique that can inhibit the expression of target messenger RNA (mRNA) and provides tremendous opportunity to investigate gene functions^28^. Principally, RNAi is an idiosyncratic gene silencing mechanism that employs double-stranded RNA (dsRNA) to degrade precise mRNAs^29^. This technique can be extensively used in crop protection; however, it is still limited to the laboratory^30–39^. The effectiveness of RNAi varies between species, selection of target genes, and the mode of dsRNA delivery^40,41^. The RNAi technology could efficiently silence the targeted genes in the form of feeding and transgenic plants^37,42,43^. Therefore, it is of vital importance for the management of pests resistant to pesticide or those having hidden nature such as *R. ferrugineus*.

The present study addressed several issues beginning with the characterization and temporal expression profile of the *RfVg* gene transcript and use of *RfVg*-based RNAi. Finally, RNAi application was investigated using quantitative real-time polymerase chain reaction (qRT-PCR), sodium dodecyl sulfate polyacrylamide gel electrophoresis (SDS-PAGE), and observation of oogenesis, ovarian development, fecundity, and eggs’ hatchability. Silencing of *RfVg* gene significantly reduced its expression, which lead to the failure of *R. ferrugineus* reproduction confirmed by oogenesis, ovarian development, fecundity, and egg hatchability. The RNAi results demonstrate that knockdown of *RfVg* gene has the potential to halt reproduction in *R. ferrugineus*, which warrants the development of novel control strategies against the pest.

## Results

### Full-length sequencing of *RfVg* and structural analysis

**Figure 1:**
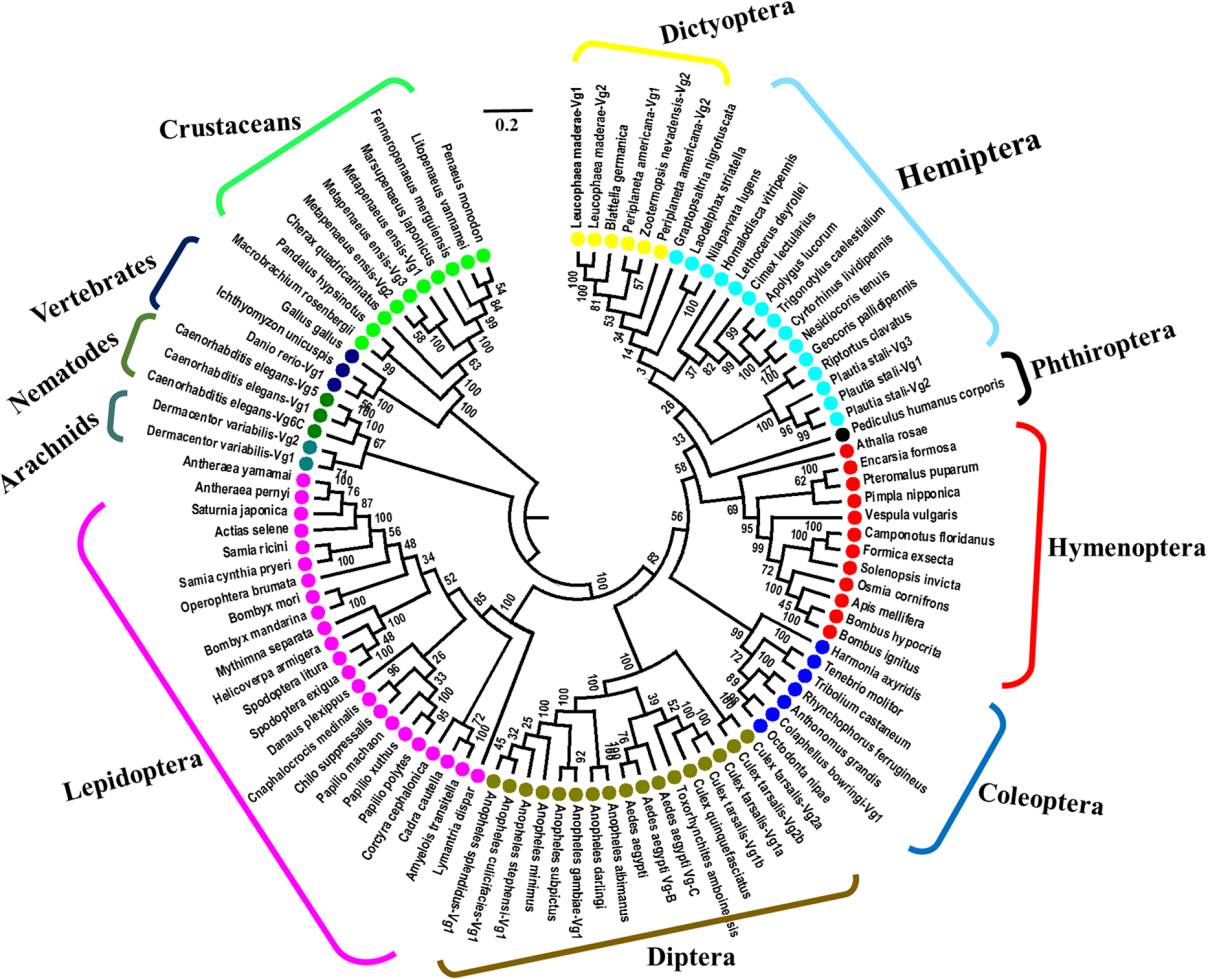
Neighbor-joining phylogenetic tree of 99 insects and non-insect Vg protein sequences. The Clustal W program was used for distance analysis of amino acid sequences and the neighbor-joining tree was constructed by using MEGA 6 software program^74^. Scale 0.2 indicates distance (number of amino acid substitutions per site)^45^ Species belonging to different orders have been indicated with different colors.

The complete *RfVg gene* transcript was of 5504 bp, which encoded 1787 residues mature protein representing all conserved structures typical of insects’ Vgs. In the Vg protein analysis, first twenty amino acid residues were predicted as signal peptide [analyzed with the SignalP program (www.cbs.dtu.dk/services/SignalP/)]. The deduced *R. ferrugineus* Vg protein contained five putative cleavage recognition sites, i.e., RRSR (361–364), RSRR (362–365), REGR (593–596), RLAR (666–669) and RPQR (1679–1682). In addition, all conserved motifs such as DGXR and GL/ICG, which are usually found at C-terminus region of Vgs also existed in the Vg of *R. ferrugineus*. The sequence of DGXR motif was DGKR (amino acids position 1,622–1,625), while of GL/ICG motif was GLCG (amino acids position 1,641–1,644). The *RfVg* contained 27 cysteine residues, of which seven were located at the C-terminus. As predicted by the NetNGlyc 1.0 program (www.cbs.dtu.dk/services/NetNGlyc/), *RfVg* contained 10 putative glycosylation sites (NXT/S). Moreover, *RfVg* contained 149 putative phosphorylation sites, including 90 serine (S), 26 threonine (T) and 33 tyrosine (Y). Moreover, three conserved domains were identified in the *RfV*g by using NCBI conserved domain database (CDD) search (www.ncbi.nlm.nih.gov/Structure/cdd/wrpsb.cgi). Among these conserved domains, one was Vg_N spanning from amino acids 21 to 735. The second domain was unknown function 1943 (DUF1943) spanning from amino acid residues 769 to 1059. The third was Von Willebrand factor domain (VWD) located at the C-terminus and spanned from amino acid residues 1467 to 1657 (Fig. S1). To elucidate the evolutionary relationship of *RfVg*, a neighbor joining phylogenetic tree was generated based on 99 insect and non-insect Vgs sequences present in the NCBI database (Fig. 1). The sequence of *RfVg* was grouped with other coleopteran Vgs as expected. The evolutionary relationship based on the current phylogenetic tree analysis showed that Vgs from insects are closer to the Vg of nematode and arachnids as compared to Vg of vertebrate and crustacean.

### Expression pattern and developmental traits of *RfVg* gene

**Figure 2:**
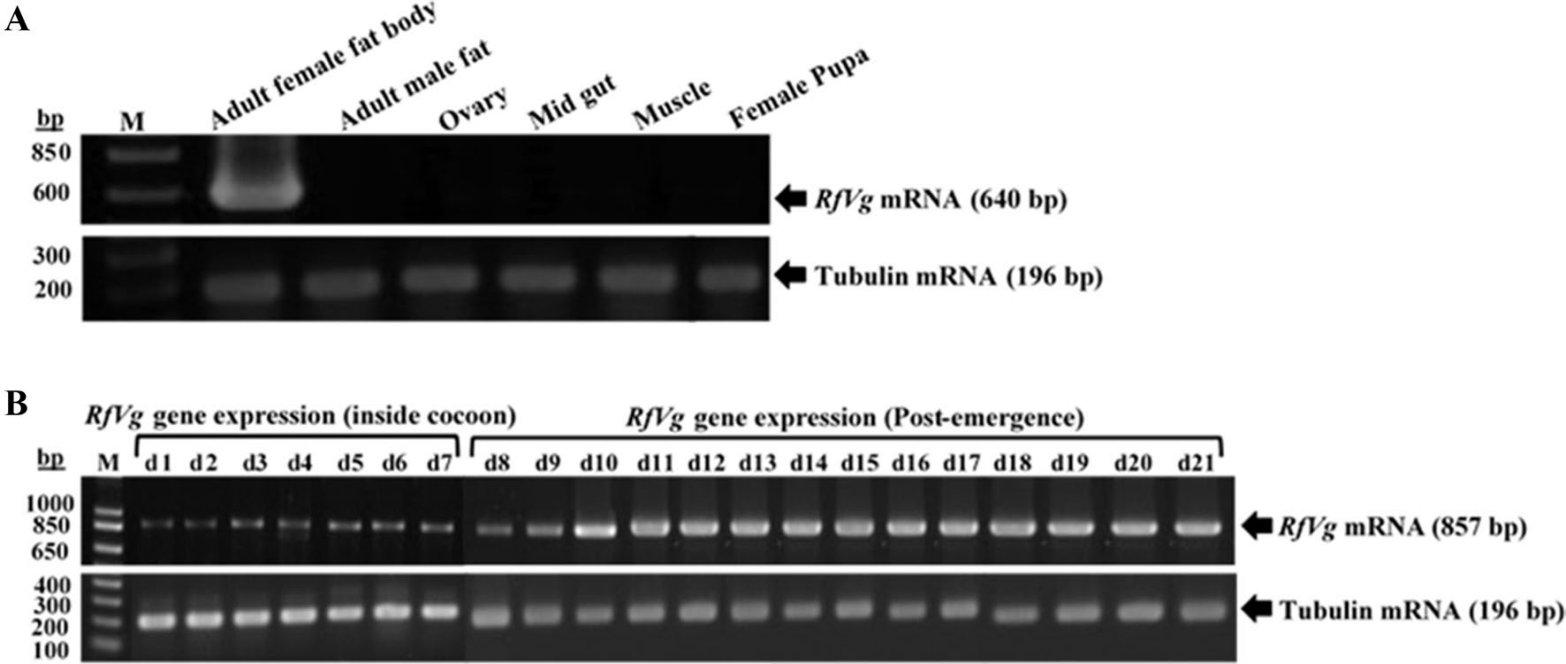
Expression pattern and temporal traits of *RfVg* gene transcription. **(A)** Expression pattern of *RfVg* and *tubulin* genes from different tissues of *Rhynchophorus ferrugineus* was analyzed by RT-PCR. Agarose gels (2%) were used to analyze the amplified PCR products. The M is a molecular marker (bp), while 640 bp and 196 bp on right side are amplified products of *Vg* and *tubulin* genes, respectively. **(B)** The *RfVg* and *tubulin* genes expression profile up to three weeks in the adult *R. ferrugineus* females analyzed by RT-PCR. The amplified bands were visualized under the UV light and photographed by using gel documentation BioDocAnalyze system (Biometra). *d*  days.

The RT-PCR was conducted to determine the sex, tissue, and stage-specific expression and probe the temporal profiles of *RfVg* gene transcription. The RT-PCR studies were performed by amplifying *RfVg-*specific region by using gene-specific primers (*RfVg*RTF2, *RfVg*RTR2) (Table S1). The *RfVg* gene was exclusively expressed in the female fat body cells as demonstrated by a single band, whereas no expression was observed for other tissue (Fig. 2A). The expression of *tubulin* gene -an internal control- in all tissues confirms the quality of the cDNAs used in these studies. To analyze the temporal expression profile of *RfVg* gene, total RNAs were extracted from the fat body of adult *R. ferrugineus* females up to three weeks. The RT-PCR was conducted to determine the developmental profile of *RfVg* gene transcription. The expression of *RfVg* gene was detected from the first day of *R. ferrugineus* female adults, which was still present inside the cocoons but with light bands. The expression of *RfVg* gene and intensity of the bands gradually increased after the emergence of adult females from the cocoons. The expression level of *RfVg* gene remained almost the same from day 10 to 21(Fig. 2B).

### Silencing of *RfVg* gene and qRT-PCR validation

**Figure 3:**
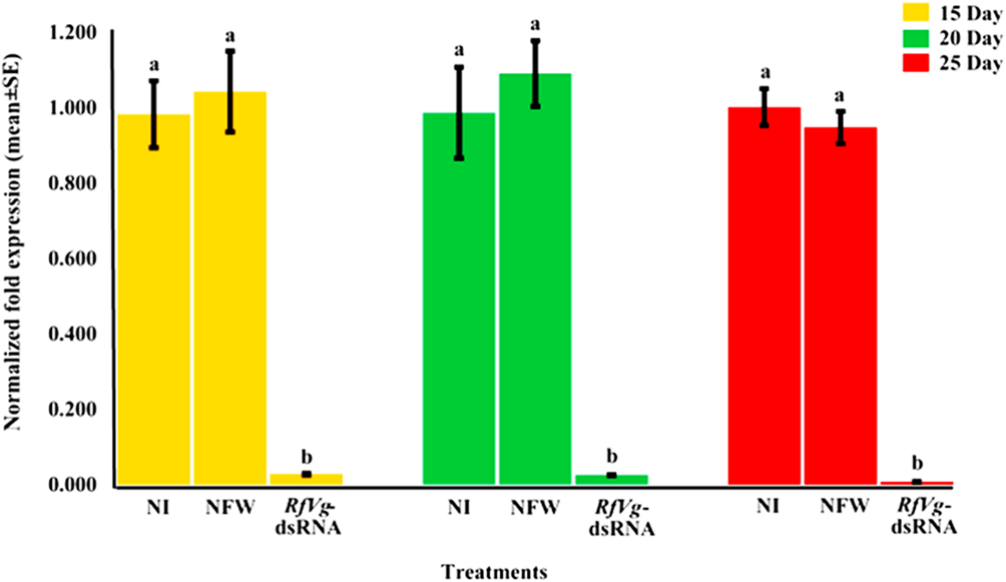
The RNAi-based silencing of *RfVg* gene results in significant down regulation of *Vg* gene. Expression levels of the *RfVg* gene were quantified by quantitative real time PCR. Normalized fold expression of the *RfVg*-dsRNA-injected group were compared with no injection (NI) and nucleus free water (NFW) groups on day 15, 20, and 25 of post-injection periods. Different letters above the bars **(a,b)** show significant differences among the groups at (α = 0.05). The expression levels of *RfVg* gene were significantly reduced in *RfVg*-dsRNA injected group in comparison to the NI injection and NFW injected groups. All biological groups contained three replicates. Each replicate has a single animal, while there were three technical replicates.

For RNAi-mediated silencing of *RfVg* gene function, dsRNA targeting an inimitable region (position 3538–3938 bp) showing very low or no homology with other insect Vgs was injected (2-μg/weevil) dorsally in the second abdominal segment of the female pupae. The qRT-PCR was executed to validate the impact of Vg-based RNAi on *Vg* gene transcription. *Tubulin* was used as an endogenous gene to fulfill the requirements of 2^−∆∆CT^ calculation method. The qRT-PCR revealed that the level of *Vg* gene expression was drastically declined in *RfVg*-dsRNA injected females as compared to nucleus free water (NFW)-injected and no injection (NI) groups (Fig. 3). In RNAi-treated females, the *Vg* gene expression was suppressed by 95.3% 15 days after injection, whereas 96.6 and 99.4% downregulation in *RfVg* gene expression was recorded on 20 and 25 days post-injection, respectively.

### Validation of *RfVg* gene silencing through SDS-PAGE

**Figure 4:**
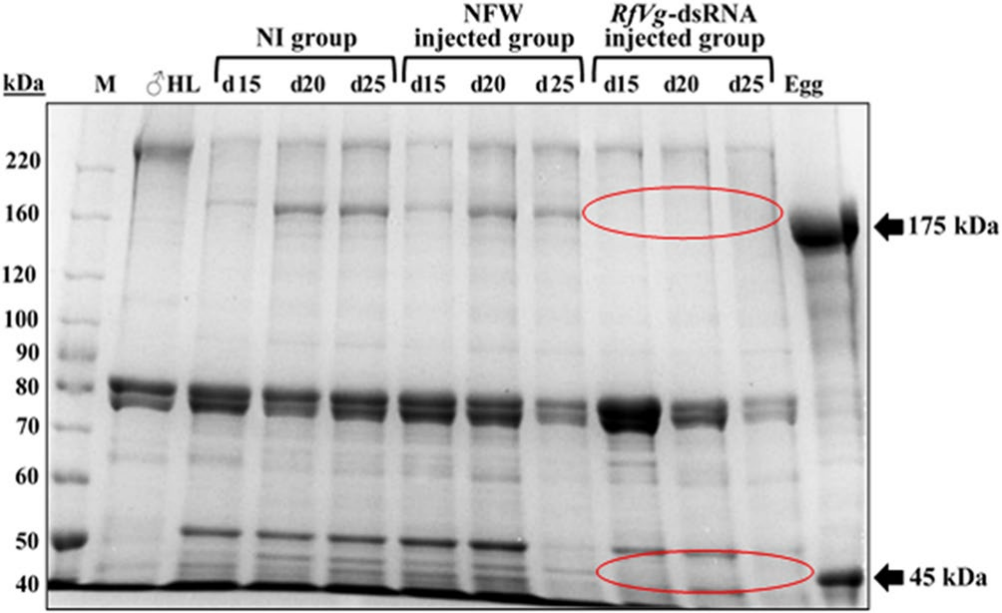
Validation of RNAi and expression analysis of *RfVgs* by SDS-PAGE. To clarify the RfVg protein expression, hemolymph samples (10 μl/lane) prepared from NI, NFW, and *RfVg*-dsRNA injected groups on 15, 20, and 25 days (d) post-injection periods were loaded on SDS-PAGE (8%) and compared. All biological groups contained three replicates; each replicate was an individual *R. ferrugineus*, while there were three technical replicates. The gel was stained with Coomassie blue and washed with destaining solution. M is the molecular marker (kDa) while, arrows on right side indicate the identified Vg polypeptides (175 and 45 kDa) in *R. ferrugineus*. A highly downregulated expression of Vg protein in *RfVg*-dsRNA injected group as compared to NI and NFW injected groups has been shown with the red circles. The protein bands were visualized under the white light and photographed by using gel documentation BioDocAnalyze system (Biometra). *d*  days.

A drastic reduction in Vg protein expression was observed in *RfVg*-dsRNA-treated females as compared to NFW and NI groups. The SDS-PAGE was executed to assess the impact of Vg-based RNAi on the expression pattern of Vg protein. The SDS-PAGE exhibited an obvious difference in expression of the Vg protein among *RfVg*-dsRNA, NFW, and NI groups (Fig. 4). Both identified Vg bands (~ 175 kDa and ~ 45 kDa) were observed in NI and NFW groups, while these bands were almost missing in *RfVg*-dsRNA-injected group.

### Biological studies to assess the impact of *RfVg* gene silencing in knockdown phenotypes

**Figure 5:**
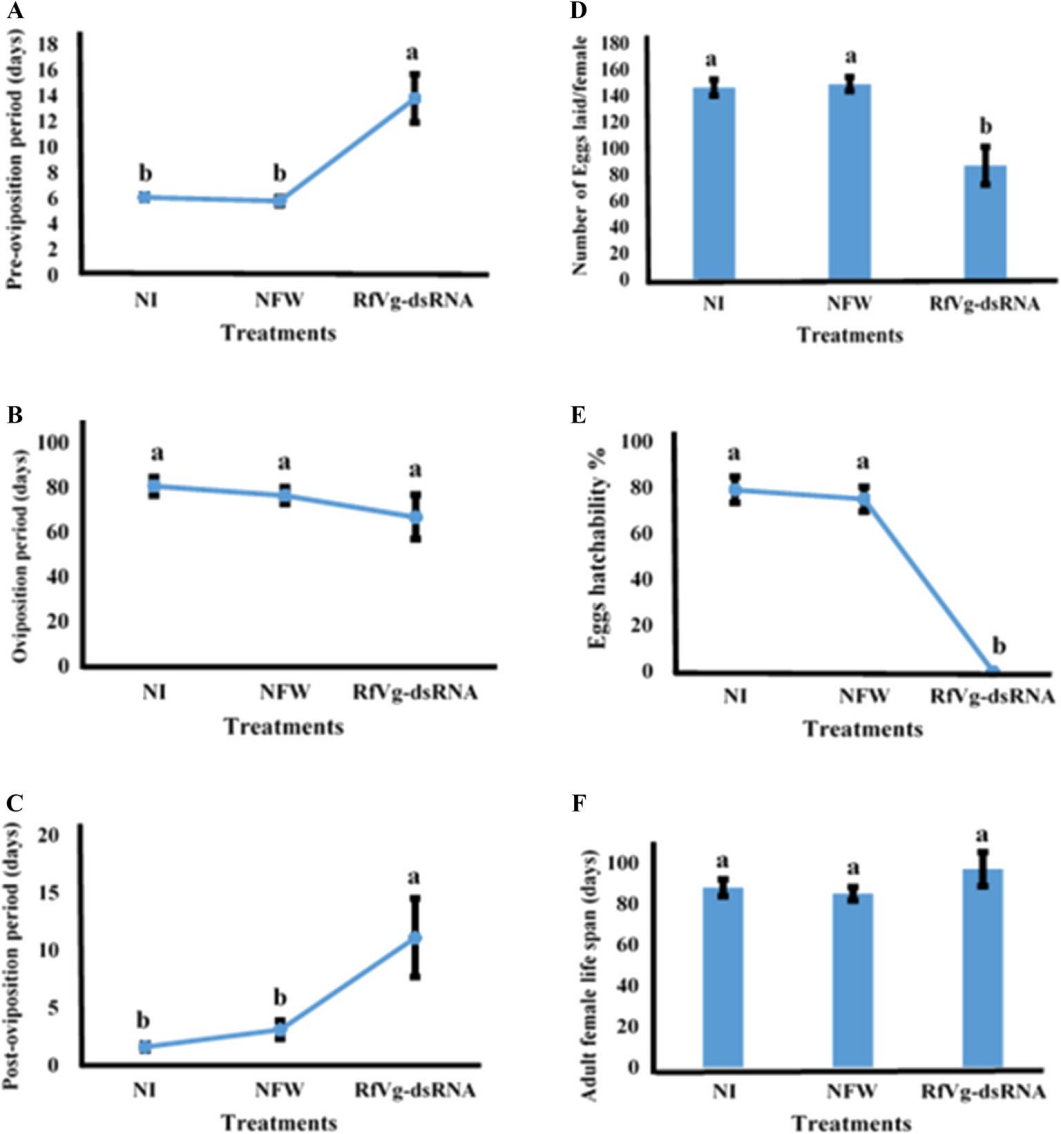
Effects of *RfVg* RNAi on reproductive traits of adult females of *Rhynchophorus ferrugineus*, pre-oviposition **(A)**, oviposition **(B)**, post-oviposition periods **(C)**, mean number of eggs laid per female **(D)**. Effects of *RfVg*-dsRNA injection on eggs hatchability % **(E)**. Effects of *RfVg*-dsRNA injection on adult female life span **(F)**. To test the statistical significance among different biological parameters, one-way analysis of variance was performed at (α = 0.05). Different letters above the bars show significant difference among groups.

The phenotypic reflection of *Vg* gene knockdown in adult females was evaluated by analyzing pre-oviposition period to assess a possible delay in egg laying, oviposition period, post-oviposition period, total number of eggs laid per female, eggs hatchability percentage, and female lifespan (Fig. 5A–F) in addition to ovarian development. The *RfVg* knockdown greatly influenced pre-oviposition period in dsRNA-treated females as compared to control groups. The data represented that pre-oviposition periods were significantly different and delayed in dsRNA-treated females (df = 2, F = 14.9, P ˂ 0.0001) in *RfVg*-dsRNA-treated females as compared to the females in NFW and NI groups (Fig. 5A). Likewise, a significant difference (df = 2, F = 6.08, P ˂ 0.007) was recorded in the post-oviposition period of RNAi-treated females and those in NFW and NI groups (Fig. 5C). A significant difference was also noted in total number of eggs laid per female (df = 2, F = 13.3, P ˂ 0.0001) where *RfVg*-dsRNA-injected group laid lesser number of eggs as compared to NFW and NI groups (Fig. 5D). Moreover, no eggs hatchability (or 0%) was observed in *RfVg*-dsRNA-injected group, whereas NFW and NI groups recorded 75 and 79% eggs hatchability, respectively (Fig. 5E). However, non-significant differences were recorded for oviposition period (df = 2, F = 1.2, P = 0.3) (Fig. 5B) and adult female lifespan (df = 2, F = 1.1, P = 0.3) (Fig. 5F).

**Figure 6:**
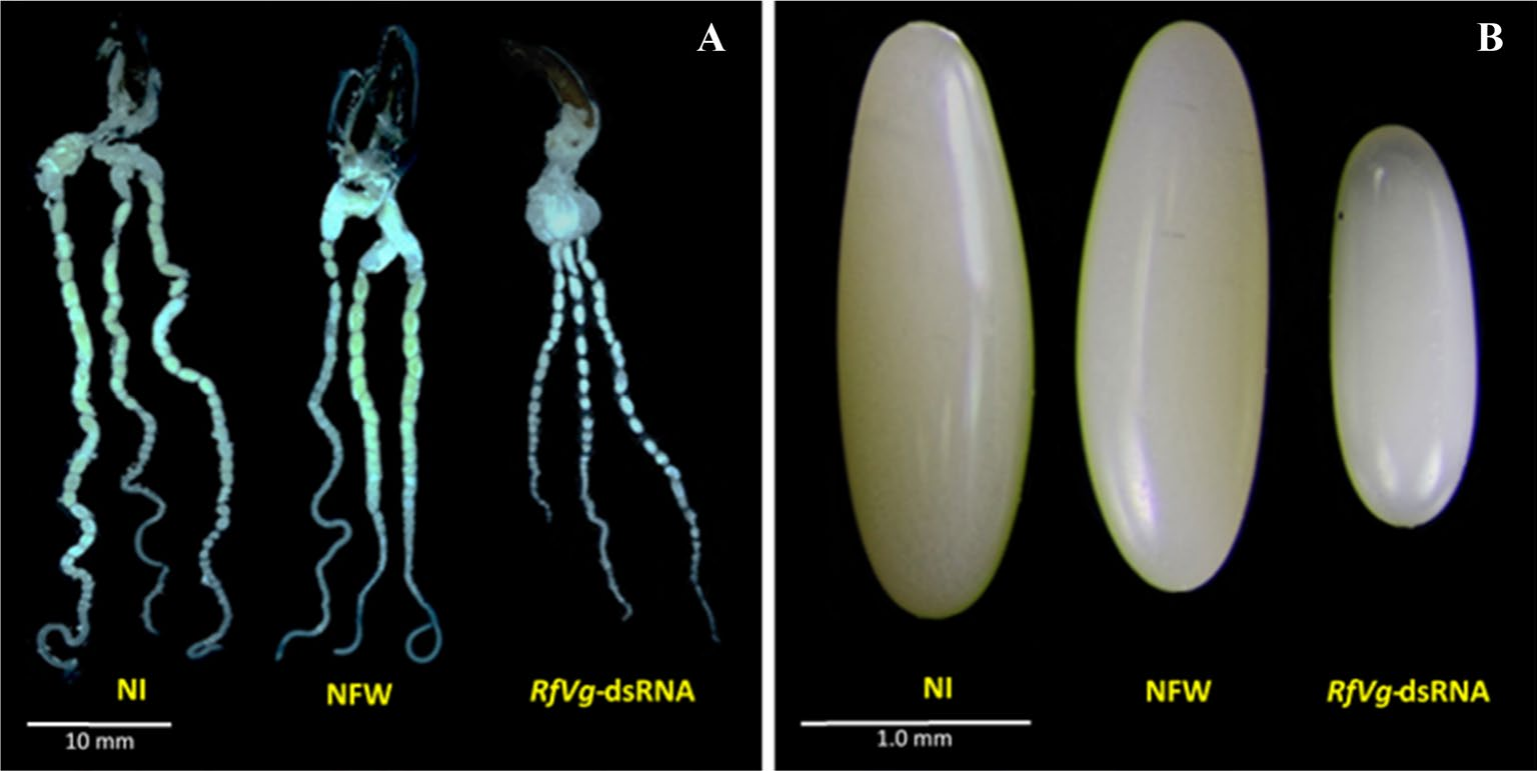
Phenotypic effects of *RfVg*-based RNAi on ovarian/egg development of *Rhynchophorus ferrugineus*. **(A)** To observe the impact of RNAi on ovarian development, ovaries from three biological groups (*RfVg*-dsRNA, NFW, and NI) were dissected 20 days post injection period, observed under a stereomicroscope (DM 165 C, Leica, Wetzlar, Germany) and compared by using auto-montage software system (Syncroscopy, Cambridge, UK). **(B)** The eggs laid by NI, NFW, and *RfVg*-dsRNA-injected females were observed under the stereomicroscope (Leica MZ 125, Germany), and compared by using the auto-montage software (Helicon focus 6). Scale bars: 10 mm and 1.0 mm for ovaries and eggs, respectively.

The investigations on ovarian development in *RfVg*-dsRNA-injected females also revealed remarkable phenotypic repercussions. The ovaries were decreased with lesser yolk compared the ovaries from NFW and NI groups where oocytes were well-developed and ovaries were larger in size (Fig. 6A). Moreover, a drastic decrease in the egg size was observed in RNAi-treated females as compared to the control groups (Fig. 6B).

**Figure 7:**
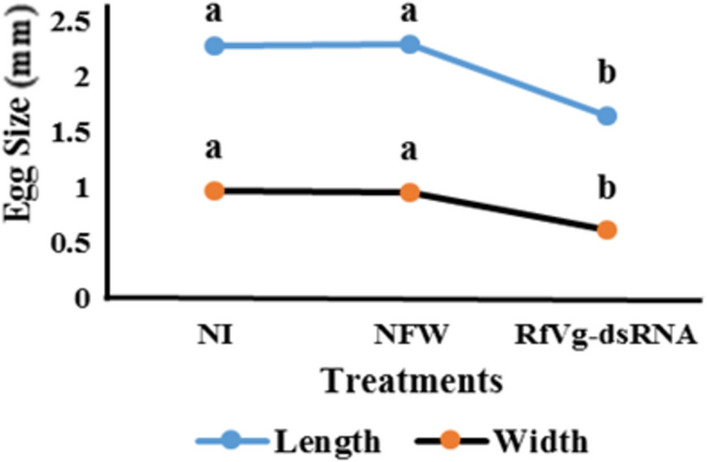
Effects of *RfVg*-based RNAi on eggs size of *Rhynchophorus ferrugineus*. To identify the impact of *RfVg* based RNAi on egg size, 15 eggs from each group (NI, NFW, and *RfVg*-dsRNA) were measured by using Dino-Lite Digital Microscope AM4815ZT (AnMo Electronic Corp, USA). To test the statistical significance among different biological parameters a one-way analysis of variance was performed at (α = 0.05). Different letters **(a,b)** above the bars show significant difference among the treatment groups.

The length (df = 2, F = 170.4, P ˂ 0.0001) and width (df = 2, F = 73.7, P ˂ 0.0001) of eggs significantly differed among treatment groups (Fig. 7). Overall, low egg production, delayed oviposition periods, smaller-sized eggs with no hatchability were recorded for *RfVg*-dsRNA-injected females suggesting that RNAi-targeting *Vg* gene has the ability to inhibit reproduction in *R. ferrugineus*.

## Discussion

Red palm weevil [*Rhynchophorus ferrugineus* (Olivier)] has become a noxious pest of palm trees around the world. It has gained significant importance due to its global invasion and associated economic costs. Recent molecular studies have revealed that Vg structures and functions seemed conserved across diverse insect taxa, although some deviations exist in post-translational processing/number of cleavage sites, number of *Vg* genes, and in the hormonal system regulating these genes^6,44,45^. The Vgs proteins have been sequenced and characterized from several insect species representing different orders, including dictyoptera^14,15,25,46^, hemiptera^27^, lepidoptera^16^, hymenoptera^47,48^, diptera^49^, and coleoptera^50–52^ due to their prime importance in insect reproduction. However, there is no information available on molecular mechanism of *R. ferrugineus* reproduction. The present study, therefore, focused on molecular characterization, expression profiling, and silencing of the reproduction control gene *Vg* in *R. ferrugineus*.

The *RfVg* gene transcript comprised of 5,504 bp nucleotides that encoded deduced protein of 1,787 amino acids. The molecular weight of RfVg protein was 210 kDa, which is almost similar to other insects’ Vgs, including coleopterans^50,51,53^. Like other coleopterans, conserved domains were present in *RfVg* (Fig. S1). Conserved domains play critical role in organisms’ physiology^54,55^. The RfVg protein have 5 post-translational cleavage sites without polyserine clusters, which shows robust structural similarity with known Vgs of coleopteran^50–52^ and some other insects including cotton leafworm (*Spodoptera litura*)^16^, parasitoid wasp (*Encarsia formosa*)^56^, and fire ant (*Solenopsis invicta*)^57^. The precursor Vg in most of the insects are post-translationally modified and proteolytically cleaved at a consent cleavage site (RXXR) into subunits by dibasic endoproteases^58^. Moreover, presence of 149 phosphorylation sites (S, T and Y) in RfVg protein sequence (Fig. S1) indicated that RfVg is highly phosphorylated similar to several other insects^50,51,59^. In addition, the presence of GL/ICG motif and cysteine residues at C-terminus are essential for oligomerization^60,61^. Furthermore, this study first time reports the data, which support potential use of *RfVg* gene silencing as a tool for the management of *R. ferrugineus*. The gene silencing approach has been successfully demonstrated against several specific genes in targeted insects, including *Vg* using different delivery methods, i.e., injection, feeding, and drops^30–39,62,63^.

The results of *RfVg*-based RNAi revealed a drastic decrease in the expressions of *Vg* gene in *RfVg*-dsRNA group as compared to females in NFW and NI groups (Fig. 3). Almost 95.3% suppression was noted in the expressions of *Vg* mRNA for *RfVg-*dsRNA injected group 15 days after injection, whereas 96.6% and 99.4% suppression was recorded on 20 and 25 days after injection, respectively. These findings clearly demonstrate that expressions of *RfVg* gene in *R. ferrugineus* females was strongly affected by *RfVg*-dsRNA treatment. The drastic reduction in *Vg* mRNA level also confirm the sensitivity of *R. ferrugineus* to RNAi. Generally, literature indicates that coleopteran species are more sensitive to RNAi than other insect groups as shown in western corn rootworm (*Diabrotica virgifera*)^37^, red flour beetle (*Tribolium castaneum*)^64^, Colorado potato beetle (*Leptinotarsa decemlineata*)^65^, and cotton boll weevil (*Anthonomus grandis*)^62^. For example, *Vg* gene expressions in cotton boll weevil were reduced by 97% just within 72 h after injection^62^. The persistence of *RfVg*-dsRNA was examined on 15, 20, and 25 days after injection in the current study in addition to reproduction performance and a drastic reduction in *RfVg* expression (99%) was observed within 25 days after injection. Likewise, results of western corn rootworm neonates fed on V-ATPase-A gene based transgenic plants indicated the silencing of a particular gene, which lead to death of treated neonates^37^. Likewise, feeding of Actin and Copβ gene based dsRNA expressed bacteria to Colorado potato beetle reduced growth and caused mortality^65^. In contrast, RNAi efficiency is less supported in other insect groups, i.e., lepidopteran insect species such as light brown apple moth (*Epiphyas postvittana*) where third instar larvae were fed with carboxylesterase-based dsRNA through droplets and < 50% mRNA silencing was achieved^66^. The major factor between coleopteran and lepidoptera RNAi efficiency includes the uptake of dsRNA and its processing to siRNA. The cells and tissues in coleopteron insects uptake dsRNA quickly and process it into siRNA faster than lepidopteran species^41^. Similarly, RNAi indicated 50 and 40% efficiency against mid-gut protein tsetse EP and nitroporin-2 in *Glossina morsitans morsitans* and *Rhodnius prolixus*, respectively^67,68^.

Additionally, a striking reduction in the expressions of *RfVg* gene resulted in dramatic failure of Vg protein expression in the *RfVg*-dsRNA-injected group as compared to NFW and NI (Fig. 4). Silencing of *Vg* gene not only caused failure of Vg protein expression, but also affected the transport of other associated nutrients^63,69^.

Furthermore, present study also confirmed the consequences of *RfVg*-dsRNA on reproductive performance of *R. ferrugineus* females, where several parameters were observed, including pre-oviposition and oviposition periods, fecundity, egg hatchability, post-oviposition period, and female lifespan. No egg hatchability was recorded for *RfVg*-dsRNA-injected group, 75.2 and 78.6% egg hatchability was found in NFW-treated and NI groups, respectively (Fig. 5E). No egg hatchability was the result of significant reduction in expression level of *RfVg* gene, which caused inadequate production of vitellogenin protein to confirm normal egg size and hatchability. Moreover, this study revealed a significant difference in number of eggs laid per female among the treatment groups (Fig. 5D) in addition to no egg hatchability. Likewise, eggs’ viability was dramatically decreased (˂1%) in *Vg* dsRNA-injected females of cotton boll weevil and eggs were unable to hatch. However, injection of *Vg*-dsRNA had no effect on fecundity of this pest^62^. Moreover, in bedbug (*Cimex lectularius*) *Vg*-dsRNA injection radically suppressed egg production compared to control group and egg production was entirely ceased two weeks after *Vg*-based dsRNA injection^63^. Additionally, results of *Vg* dsRNA injection also decreased the eggs production in lubber grasshopper (*Romalea microptera*)^69^.

The pre-oviposition periods were delayed in dsRNA-treated females. The effect of *Vg* knockdown on pre-oviposition period has been reported earlier^63^; however, no effect of RNAi on pre-oviposition period of cotton boll weevil has also been reported^62^. Besides, present study revealed that ovaries were rigorously deformed with short and unorganized eggs and ovary size (Fig. 6A,B). The size of eggs was significantly decreased in *RfVg*-dsRNA-treated group as compared to NFW and NI groups (Fig. 7). Similarly, eggs development in cotton boll weevil was severely affected in *Vg* dsRNA-injected group^62^. Moreover, results of RNAi injection revealed shrunken ovaries in bedbug with no developed oocytes in *Vg* dsRNA-treated female as compared to control where ovaries were normal in size with mature oocytes^63^. Likewise, ovaries were rigorously deformed in rice moth having small ovarioles and disorganized egg sizes in *Vg* dsRNA-treated females relative to un-injected females^31^.

The present findings along with previous reports conclusively demonstrate a high potential of Vg-based RNAi technology for pest management. Silencing of *RfVg* gene provides evidence that RNAi technology could be a smart alternative to traditional management methods for coleopteran pests, particularly for *R. ferrugineus*. Undoubtedly, the success and effectiveness of RNAi varies with species, selection of target genes, and the mode of dsRNA delivery^40,41,70^. The present study has provided evidence that *Vg* gene is the best target for RNAi-based management of *R. ferrugineus*. Choosing a suitable tactic to deliver the dsRNA successfully after selection and identifying the target gene is a main challenge in RNAi-based plant protection method. Although microinjection is a suitable approach for functional genomic studies, this strategy is not appropriate to manage the pest in the field. However, numerous developments on dsRNA delivery made this technique more efficient in the field. For example, delivering dsRNA to insect pests through transgenic plant has been tried^37^. Moreover, successful feeding of insects pest via bacterially expressed dsRNA^65^ and application of dsRNA through nanoparticles^71^ have also been practiced. The silencing of *RfVg* gene by RNAi may have the potential to stop the reproduction of *R. ferrugineus* and *RfVg* could be an auspicious target candidate gene for developing an alternative pest management strategy for the pest at molecular level. Therefore, the future research should be focused on the delivery of *RfVg*-dsRNA for the management of *R. ferrugineus* in the field. This study supports the potential use of emerging RNAi technology for pest control and might provide an alternative to the conventional methods being used for the management of *R. ferrugineus*.

## Materials and methods

### Rearing of the red palm weevil

Red palm weevils were originally collected from infested date palm trees in Dirab, Kingdom of Saudi Arabia (24.4164°N, 46.5765°E). The adults were provided a piece of cotton saturated with 10% sugar solution^72^ and kept in plastic box (L: 17 cm; W: 11 cm; H: 7 cm). The laid eggs were collected with the help of forceps and shifted to wet filter papers placed in small plastic cup (d: 6 cm; h: 2.5 cm). The larvae were fed with artificial diet (250 g/5 larvae) for further development in the plastic box (L: 17 cm; W: 11 cm; H: 7 cm). Finally, the last instar larvae were shifted into a sugarcane set (10 cm) for pupation in plastic boxes (L: 17 cm; W: 11 cm; H: 7 cm). The R*. ferrugineus* culture was maintained in the growth chamber at 25 ± 1 °C, 70 ± 5% relative humidity^72^.

### PCR amplification and sequencing to obtain full length *RfVg* and phylogenetic analysis

The partial sequence of *RfVg* gene transcript was obtained through the next-generation sequencing (NGS) of *R. ferrugineus* fat body tissues from Beijing Genomics Institute (BGI), China. The gene-specific primers (*RfVg*F1), which were designed based on the partial *RfVg* sequence and the adopter primer 1 (AP1) (Clontech) (Table S1) were used for 3ʹ RACE-PCR in order to get the full-length sequence of *RfVg* gene. The ds cDNA library was subjected to PCR by using the Gene Amp PCR system 9700 thermo cycler (Applied Biosystems, USA). The PCR conditions were; initial denaturation at 94 ºC for 1 min followed by 35 cycles of denaturation at 94 ºC for 30 s, annealing at 68 ºC for 3 min, and a final extension of 68 ºC for 5 min. The amplified PCR products were purified by using illustra GFX PCR DNA and Gel Band Purification Kit (GE Healthcare Life Sciences, USA). The purified PCR products were sequenced from BGI, China. The obtained sequences were analyzed and checked for homology with other insects *Vg* sequences by using basic local alignment search tool (BLAST) of National Center for Biotechnology Information (NCBI). Finally, *RfVg* sequence was submitted to the NCBI GenBank database (accession number ALN38803) after confirmation. The *RfVg* sequence was aligned to other known insects Vgs sequences available in the NCBI database using the clustalW program^73^. Phylogenetic tree was constructed using neighbor-joining method on MEGA version 6^74^.

### Expression pattern and developmental traits of *RfVg* gene

To investigate the tissue, gender-specific expression, and developmental profile of *RfVg* gene transcription, total RNAs were extracted from the female fat body, ovary, mid-gut, muscle, male fat body, and female pupa. Total RNA was extracted from fat body of adult *R. ferrugineus* females up to three weeks (for one week when the female adults were inside the cocoons and two weeks after the eclosion) by using Tri-RNA reagent (Favorgen Biotech Corp, Taiwan) to analyze the developmental expression profile of *RfVg* gene.The RNA samples were treated with DNase I (Invitrogen, USA) to remove DNA contamination. A 2-μg of total RNA from each sample was reverse transcribed to cDNA using ReverTra Ace cDNA synthesis kit (Toyobo Co. Ltd, Japan). The reverse transcription polymerase chain reaction (RT-PCR) was performed by using gene-specific primers (*RfVg*RTF1, *RfVg*RTR1, *RfVg*RTF2, *RfVg*RTR2) and *tubulin* was used as internal control (*TubulinRfer*-F, *TubulinRfer*-R) (Table S1). The cDNA was subjected to RT-PCR by using Gene Amp PCR system 9700 thermo cycler (Applied Biosystem, USA). The following thermal programs were applied; 94 ºC for 1 min for denaturation followed by 30 cycles of 94 ºC for 30 s, 68 ºC for 30 s, and 72 ºC for 1 min. The PCR amplified products were run on 2% agarose gel, stained with ethidium bromide, and visually confirmed under ultra violet (UV) light by using gel documentation BioDocAnalyze system (Biometra, Germany).

### RNAi-based silencing of *RfVg* gene and tissue collection for RNAi validation

A unique target region of 400 bp showing very low or no homology with other insect Vgs was selected from the C-terminus of the *RfVg* gene transcript. The *RfVg*-based dsRNA was synthesized using a MEGAscript® RNAi Kit (Life Technology, USA) according to the manufacturer’s protocol and used to knockdown the function of *RfVg* gene. The experiment consisted of three biological treatment groups, which were *RfVg*-dsRNA-injected, nuclease free water (NFW)-injected, and no injection (NI)^31,39,76–78^. A total 2-μg (10-μl) of *RfVg*-dsRNA was injected dorsally in the second abdominal segment of each 10–12 days-old female pupae by using 0.5 ml BD Micro-Fine^tm^ Plus syringe (Becton, Dick-inson Co, USA), whereas 10-μl of NFW was injected as control. The pupae used for injection were taken from the cocoons, which were placed again in the cocoon after injection for further development and kept in growth chamber at 25 ± 1 °C, 70 ± 5% relative humidity and photoperiod of 12:12 (L:D)^5^. When pupae converted to adults, they remained inside the cocoon for almost one week before eclosion. The female pupae were selected on the base of snout as *R. ferrugineus* female has smooth narrow long snout, while male has shorter and wider snout with some tuft hairs. Furthermore, females were also confirmed after adult eclosion. The newly emerged females (from cocoon) were shifted to a separate box having a piece of cotton saturated with 10% sugar solution. The females were harvested on three post-injection periods (i.e., 15, 20, and 25 days) for the collection of fat body (for cDNA synthesis to analyze *Vg* transcript levels), hemolymph (for Vg protein expression analysis) and ovaries. The RNAi experiments were validated using quantitative real time polymerase chain reaction (qRT-PCR), sodium dodecyl sulfate polyacrylamide gel electrophoresis (SDS-PAGE), and by observing phenotypic effects of RNAi on ovarian development. The cDNA and hemolymph samples were prepared for all biological groups. Total RNAs were extracted from the fat body by using Tri-RNA reagent (Favorgen Biotech Corp, Taiwan) to make cDNA. The RNA samples were treated with DNase I (Invitrogen, USA) to remove the DNA contamination. A 2-μg of total RNA from each sample was reverse transcribed to cDNA using ReverTra Ace cDNA synthesis kit (Toyobo Co. Ltd, Japan). Hemolymph was collected with the help of micropipette after the snout of weevil was amputated using a fine scissors and diluted to 1:50 with the sample buffer.

### Validation of *RfVg* gene silencing through qRT-PCR

A qRT (quantitative real time)-PCR*,* analysis was performed by using the *RfVg*-gene-specific primers (*RfVg*RTF3 and *RfVg*RTR3) to corroborate the impact of *RfVg*-based RNAi on *Vg* gene expression (Table S1). Expression levels of the *RfVg* gene were normalized by quantifying the expression levels of *tubulin* (a housekeeping gene) using *TubulinRfer-*F and *TubulinRfer*-R primers (Table S1). The qRT-PCR trials were designed based on three biological groups, which were *RfVg*-dsRNA, NFW, and NI. All groups contained three replicates, each replicate had a single animal, while there were three technical replicates. The qRT-PCR was accomplished using CFX-96 Touch™ Real-Time PCR Detection System (BioRad, USA), while reactions (each contained a volume of 20-μl) were performed using SsoAdvanced™ Universal SYBR^®^ Green Supermix (BioRad, USA). The following qRT-PCR conditions were applied for amplifying the cDNA; 95 ºC for 30 s, 40 cycles of 95 ºC for 15 s, 60ºC for 60 s, followed by melting curve analysis at 65–95 ºC with an increment of 0.5 ºC every 5 s. The 2^−∆∆CT^ method was used to analyze the relative expression levels of *RfVg* gene by normalizing them to *tubulin* and control (NI) group.

### Validation of *RfVg* gene silencing through SDS-PAGE

The Vg protein expression levels in dsRNA-injected females were analyzed through SDS-PAGE as reported previously^15,27^ to affirm the efficiency of RNAi. Protein analyses were conducted using samples of hemolymph and egg extracts (each 10 μl) run on 8% polyacrylamide gels. Three post-injection periods, i.e., 15, 20 and 25 days (after injection with dsRNA) with three biological groups (*RfVg*-dsRNA, NI, and NFW) were analyzed. All groups contained three replicates; each replicate was an individual of *R. ferrugineus*, while there were three technical replicates. The Vg protein expression levels in *RfVg*-dsRNA injected-weevils were compared with the NI and NFW groups. The gels were stained with Coomassie blue and washed with de-staining solution. The protein bands were visualized under the white light and photographed by using gel documentation BioDocAnalyze system (Biometra, Germany).

### Biological studies to assess the impact of *RfVg* gene silencing in knockdown phenotypes

The phenotypic manifestation of *RfVg* gene silencing in RNAi-treated *R. ferrugineus* females was assessed based on the biological markers including pre-oviposition periods, oviposition periods, total number of eggs laid per female, eggs hatchability %, post-oviposition periods, and female life span in addition to the ovarian development. To inspect these biological traits, newly emerged adult females (dsRNA-injected) were paired with the normal males, transferred to a separate plastic box (1 kg) having a piece of cotton saturated with 10% sugar solution and kept in growth chamber at 25 ± 1 °C, 70 ± 5% relative humidity^72^. There were again three biological groups, i.e., *RfVg* dsRNA-injected (dsRNA), NFW-injected, and NI. All groups contained nine replicates and each replicate was an individual pair (normal male + dsRNA-treated female) of *R. ferrugineus*. All pairs were allowed to mate and lay eggs until females died. The oviposition periods were observed and the number of eggs and hatchability percentage were scored. Completely randomized design (CRD) was used in this experiment.

To investigate the impact of Vg-based RNAi on ovarian development, ovaries from all three groups (*RfVg*-dsRNA, NFW and NI) were isolated in the phosphate buffered saline (PBS) 20 days post-injection periods, viewed under stereomicroscope (DM 165 C, Leica, Wetzlar, Germany) and photographed using auto-montage software system (Syncroscopy, Cambridge, UK) to measure the ovarian development. Eggs from dsRNA-injected groups were compared with NFW and NI groups, observed under the stereomicroscope and photographed (Leica MZ 125, Helicon focus 6 software, Germany). Furthermore, 15 eggs from each group (NI, NFW, and *RfVg*-dsRNA injected groups) were measured using Dino-Lite Digital Microscope AM4815ZT (AnMo Electronic Corp, USA) to clarify the impact of *RfVg* based RNAi on egg size (length and width).

### Statistical analysis

The qRT-PCR quantification results were analyzed following 2^−∆∆CT^ method (Livak and Schmittgen, 2001). One-way analysis of variance was performed at (α = 0.05) by using SAS program ver. 9.2. To analyze the statistical significant differences among three experimental groups (*RfVg*-dsRNA, NI and NFW) for qRT-PCR data and biological studies^75^.

### Ethics approval

The work is original. Moreover, no legal permission was required to conduct the experiments.

## Supplementary Information

Supplementary Information.

## Data Availability

All the data is present in the manuscript.
