# Supplementary material for: Silencing of vitellogenin gene contributes to the promise of controlling red palm weevil, Rhynchophorus ferrugineus (Olivier)
Source: Sci Rep. 2021 Nov 4;11:21695. doi: 10.1038/s41598-021-01159-9 (PMC8568968; doi:10.1038/s41598-021-01159-9)
Supplement: Supplementary file 1 — Supplementary Information. [file 41598_2021_1159_MOESM1_ESM.pdf]

```
     atgtggtcgccgctagcattactgttcctggtgggatatgctctcgcttcaacaccggct     60
 20   M  W  S  P  L  A  L  L  F  L  V  G  Y  A  L  A  S  T  P  A
     tttaaggacaacaccgaatatgtttacgatgtcaacggacgaactctcagtagtttacat    120
 40   F  K  D  N  T  E  Y  V  Y  D  V  N  G  R  T  L  S  S  L  H
     gaagtttccgaccagtattccggtattttcctaaaagcaaaactgcacctgagcaaacga    180
 60   E  V  S  D  Q  Y  S  G  I  F  L  K  A  K  L  H  L  S  K  R
     tctgacgacaaagtccaaggacggatttctgaccctcaatatgctccaattcatagccat    240
 80   S  D  D  K  V  Q  G  R  I  S  D  P  Q  Y  A  P  I  H  S  H
     ttatgcgacgggtgggatactgaagttcccgaatctcaattgagctacaaacaactggct    300
100   L  C  D  G  W  D  T  E  V  P  E  S  Q  L  S  Y  K  Q  L  A
     ctatctgggaaacccttcgaaattggtatggacagcgctggtcttattaaaaacatcgtt    360
120   L  S  G  K  P  F  E  I  G  M  D  S  A  G  L  I  K  N  I  V
     gttgaaaaggaagtcagtaacatggaagcaaatataatcaagagtatttgcagtcagtac    420
140   V  E  K  E  V  S  N  M  E  A  N  I  I  K  S  I  C  S  Q  Y
     cagttggatgttcgaggaaagaatgccatcgatagtccaattaatgatctcccttctgaa    480
160   Q  L  D  V  R  G  K  N  A  I  D  S  P  I  N  D  L  P  S  E
     gataaattggatggagttttcaaaaccatggaggaaacagttaccggtgagacggaaact    540
180   D  K  L  D  G  V  F  K  T  M  E  E  T  V  T  G  E  T  E  T
     acctacaaaatgcatcctttgcccttgtacattttgcaatctcaaccttggttggtacgt    600
200   T  Y  K  M  H  P  L  P  L  Y  I  L  Q  S  Q  P  W  L  V  R
     aaagacgatttgcaacaagacaacgatagagtagtcgaggtaatcaaaagcaagaactac    660
220   K  D  D  L  Q  Q  D  N  D  R  V  V  E  V  I  K  S  K  N  Y
     accaacagcgaagaaaaaccttcgtatcactacggttttggtgatatccatgaacatgaa    720
240   T  N  S  E  E  K  P  S  Y  H  Y  G  F  G  D  I  H  E  H  E
     ccaactgccaatagcttgggacaatttcttaccagacaatctcactctcgcgctattttg    780
260   P  T  A  N  S  L  G  Q  F  L  T  R  Q  S  H  S  R  A  I  L
     accggtaaaccttccagatttaccattcaaaacagttacaccgtgaacaaaattatgatc    840
280   T  G  K  P  S  R  F  T  I  Q  N  S  Y  T  V  N  K  I  M  I
     aggcccaccttcaataacaaagaaagaggttcggtgatcagtatggtgaatgtcactcta    900
300   R  P  T  F  N  N  K  E  R  G  S  V  I  S  M  V  N  V  T  L
     agagaggttaagaatcaagatcaaaaaccacaagatttatccaaccctaatgatattggt    960
320   R  E  V  K  N  Q  D  Q  K  P  Q  D  L  S  N  P  N  D  I  G
     aaccttatctacacctacgacaatcctttttctcaaaacaaagatgccaagcaaaagagg   1020
340   N  L  I  Y  T  Y  D  N  P  F  S  Q  N  K  D  A  K  Q  K  R
     atggagaagtatcactccagcgaggaacgtagtgatagcgaagaagagacatcatggggc   1080
360   M  E  K  Y  H  S  S  E  E  R  S  D  S  E  E  E  T  S  W  G
     aggagaagtcgtcgtcacatttcccaacatatccacagacaccaggaaggcaatcacgat   1140
380   R  R  S  R  R  H  I  S  Q  H  I  H  R  H  Q  E  G  N  H  D
     cctaaagatagccaacaacaacaacaaaaacccaggttggagcaagctcctagttcacca   1200
400   P  K  D  S  Q  Q  Q  Q  Q  K  P  R  L  E  Q  A  P  S  S  P
     ctccttccttactacatgggatatcacggaaaatcggttaagcaaagttcagattttgat   1260
420   L  L  P  Y  Y  M  G  Y  H  G  K  S  V  K  Q  S  S  D  F  D
     gttaaacaaaacgtgcaaaacatggcgcaagatatcgccgaaaatctgatggatcctgat   1320
440   V  K  Q  N  V  Q  N  M  A  Q  D  I  A  E  N  L  M  D  P  D
     aagatcttaaaacaggatactctgagtaaatacgtcatgctttcctctttgatgaggttg   1380
460   K  I  L  K  Q  D  T  L  S  K  Y  V  M  L  S  S  L  M  R  L
     atggataaggacgaaattaaacaagtatctgaacaattatacagtccgtctggtaaagga   1440
480   M  D  K  D  E  I  K  Q  V  S  E  Q  L  Y  S  P  S  G  K  G
     aaagatcgtttgacgtgggaggtgtaccgtgacgctctagccgtatctggaactggtcct   1500
500   K  D  R  L  T  W  E  V  Y  R  D  A  L  A  V  S  G  T  G  P
     gccttccttcacattaaggaactgatcgagtccaagaaaatcaccaaaggtgaagccgct   1560
520   A  F  L  H  I  K  E  L  I  E  S  K  K  I  T  K  G  E  A  A
     gatgtagtcgcgaccatggctaagtctgttcgtacacctaccgaagattacatgaaacaa   1620
540   D  V  V  A  T  M  A  K  S  V  R  T  P  T  E  D  Y  M  K  Q
     ttctttgaactcaccaagaactccagaattatggaagaagagaaattgaatcaaacggct   1680
560   F  F  E  L  T  K  N  S  R  I  M  E  E  E  K  L  N  Q  T  A
     gtgttggcttattgtaacttgttgtatagagtctatatgaatagaaatgaatcccacagt   1740
```

```
580  V L A Y C N L L Y R V Y M N R N E S H S
cagtaccctgtacacagttttggaagcttcaacagtagagagggtcgtaaatatgttaga  1800
600  Q Y P V H S F G S F N S R E G R K Y V R
gagaccgtcattccttattacaagcagcaacttgatcgggcgatttccgaaggtcaatca  1860
620  E T V I P Y Y K Q Q L D R A I S E G Q S
aacaaaatccacctgttcatcactgctttaggagacatcggtgacagagatatcttatcc  1920
640  N K I H L F I T A L G D I G D R D I L S
gcatttaaaccatacttggagggcacgaaacaatgctctcagttccaacggatgctgatg  1980
660  A F K P Y L E G T K Q C S Q F Q R M L M
gtttcctgcttgaggaggttggctcgtagccaacgtaatgctgcccttcccgtcttatat  2040
680  V S C L R R L A R S Q R N A A L P V L Y
aagatctatcagaacgccggagaacttcccgacgtcagaatcagcgctgcctacgttttg  2100
700  K I Y Q N A G E L P D V R I S A A Y V L
ttcagagatccacaagtcttatccgaacgacttcaaagtatggcagaaaacactcacatc  2160
720  F R D P Q V L S E R L Q S M A E N T H I
gaataccaggaacagttgaatgccgccgtcaaatccgccatcgaatcagcttctcgtttg  2220
740  E Y Q E Q L N A A V K S A I E S A S R L
gaatcacgcagccgtcataatctacgagatgctgctcagaccgctgtacctctgttgaac  2280
760  E S R S R H N L R D A A Q T A V P L L N
gacaaactttacggagcagataagagtcacatcaatttccgcgattacgtcataccggaa  2340
780  D K L Y G A D K S H I N F R D Y V I P E
atggactccgaattccatcatgactttgtggaaatcggtagccctgatagttattggccc  2400
800  M D S E F H H D F V E I G S P D S Y W P
aaagcaatgaaagcttacgcccgcggacacgtcaacgagatccctcaacaatactacgac  2460
820  K A M K A Y A R G H V N E I P Q Q Y Y D
ttcaaagccatgacctccagcatcaaggaattgttcgatgtcctctatgaaaaaaccagc  2520
840  F K A M T S S I K E L F D V L Y E K T S
gggtccaagcaagcaaagaaattgagatcccaagaacgagatggcgatagtaaatggtct  2580
860  G S K Q A K K L R S Q E R D G D S K W S
agtgccaatatcgcccaacaaatgaactacttcaaggaggaacgcgaacaattggagaac  2640
880  S A N I A Q Q M N Y F K E E R E Q L E N
tatatctacgctgaaatcgctggacttcaaagcatgtggtcatacgacaaccgaagcatc  2700
900  Y I Y A E I A G L Q S M W S Y D N R S I
gacaacttacctcaagccattcgtgaatacgaagacacttacagcaaaggaaaagaattc  2760
920  D N L P Q A I R E Y E D T Y S K G K E F
agctacaccaaactgagacaaatcaaggatctcgctctttctgtccccaccgaaatgggt  2820
940  S Y T K L R Q I K D L A L S V P T E M G
cttccgttcttgtacacctccgacaaaccggtgttagtcagatgggccggtaaaatcgaa  2880
960  L P F L Y T S D K P V L V R W A G K I E
gctagagctactccccagatttcagatggacaaaaactgagcagacctgataagatcaaa  2940
980  A R A T P Q I S D G Q K L S R P D K I K
gtcaaaattagcagcgcctttacgttcagcgttaaggatcagagccatttgtcattcgtc  3000
1000 V K I S S A F T F S V K D Q S H L S F V
acaccttgcgaccaccaaatatacattgccggtttcgacagaaacgtacaagctcatcta  3060
1020 T P C D H Q I Y I A G F D R N V Q A H L
cctctttcggctgatgtcgacatcgatgttaaagaaggcgaagccaccgtcgaatgcgaa  3120
1040 P L S A D V D I D V K E G E A T V E C E
gtccaaaacccagacaaggatgcccgtctgttgcattacagcacatggccttacacatcc  3180
1060 V Q N P D K D A R L L H Y S T W P Y T S
aaaggcgacttgatgagcaccagtccggtatcactaagacccaacacacaaattatccca  3240
1080 K G D L M S T S P V S L R P N T Q I I P
ccgaaagaaagtcaaagtagatatttcgatactcaattcggtaaaagccaaactggcatg  3300
1100 P K E S Q S R Y F D T Q F G K S Q T G M
accttccgtgcttggggtcatcatcccgtacaatccgtcaatctaggagatctcctccac  3360
1120 T F R A W G H H P V Q S V N L G D L L H
atgtacaaagccggagatatgaaaactttgttgaaatgggtttgggatcgttcttctctc  3420
1140 M Y K A G D M K T L L K W V W D R S S L
```

acacagactgaaatgtcggtcgcttatgtgccaaaccaatcgtccaccaagaaagttacc 3480
1160 T Q T E M S V A Y V P N Q S S T K K V T
ttacgtttctcccacaagaaacaatacaaggaacaaccagagaagaaggaacaagaagat 3540
1180 L R F S H K K Q Y K E Q P E K K E Q E D
ttcctcagctacagccagttgaaccagaaatgtcaaggtgaacccaagaaacgtcaagaa 3600
1200 F L S Y S Q L N Q K C Q G E P K K R Q E
aacttgcttaaatatgttgaagctggtattaacaacggccagtctcatgcgtatgaggta 3660
1220 N L L K Y V E A G I N N G Q S H A Y E V
tggatggaatttgatggtgataagaaacgccaacacggctgtggagtgactttctgcaag 3720
1240 W M E F D G D K K R Q H G C G V T F C K
agcaacgtagattctaaatctaggacgatgatttactatacaggaaatggcgatagcgag 3780
1260 S N V D S K S R T M I Y Y T G N G D S E
aaacaatgttctttggatgttaaagctaatataccgaacaccaacggtttggatctgact 3840
1280 K Q C S L D V K A N I P N T N G L D L T
gactctttgaaaaatgctccagaggctaagtatgaaatgcgtatgcagtgcggtccaaac 3900
1300 D S L K N A P E A K Y E M R M Q C G P N
gaaaatgacgcagctcaagtatcaggcaaggtgaactttaagagaagccaacaacgtaaa 3960
1320 E N D A A Q V S G K V N F K R S Q Q R K
gacagagttacccagaaaccactgtacaacgtatgcaaacgtcaaatgaaagaagggaac 4020
1340 D R V T Q K P L Y N V C K R Q M K E G N
ttccaactcccggcttgtcaaaatatgaccatcgaagcaaactacatggacgtcattcaa 4080
1360 F Q L P A C Q N M T I E A N Y M D V I Q
tgcaaatggcaatacaataacatcgataggaaatactctgatatcgttaaatcagtatat 4140
1380 C K W Q Y N N I D R K Y S D I V K S V Y
gaaggatttaaagtgtattattacccagaaaccaaaattgaaagtattgatgatcaaaag 4200
1400 E G F K V Y Y Y P E T K I E S I D D Q K
aacaacattcaggtggaggtaagatacgagcctgaggaattaagaagagtaaacgttagc 4260
1420 N N I Q V E V R Y E P E E L R R V N V S
attgctagtggcgatgaaagaactaccatttacaacgtctcccttggcagcgactacgct 4320
1440 I A S G D E R T T I Y N V S L G S D Y A
aaagcccttttggtgccacatcctgtgttccacgttaaatctcgattggttggtgtcctc 4380
1460 K A L L V P H P V F H V K S R L V G V L
caaggatggcaattacacagaccaacctgcgtcatcgaccaatccgctattcagaccttc 4440
1480 Q G W Q L H R P T C V I D Q S A I Q T F
agcaacaacacctatcctctgtctctcggaaatgattggactgttgctgttcaatacatt 4500
1500 S N N T Y P L S L G N D W T V A V Q Y I
ccccaggaagctcgtagaagagatcaaccaaaacaaccgtctgttttcgagcaactgaaa 4560
1520 P Q E A R R R D Q P K Q P S V F E Q L K
gaccaacaagaaaactatgccattcttgtacgtcaagcatccgaagacactaaagaagta 4620
1540 D Q Q E N Y A I L V R Q A S E D T K E V
atgatcaccttcaaccatgaagaaagcgagggaaaaacagttgagattaacttgaaatgc 4680
1560 M I T F N H E E S E G K T V E I N L K C
gaacaatctcgtcaacgtaagagatctggttctgatccagccgctactgtgtacatcgat 4740
1580 E Q S R Q R K R S G S D P A A T V Y I D
ggtaaacaaatacaattcaccgacaagcagagctatgatttatacaacggttttgtgcaa 4800
1600 G K Q I Q F T D K Q S Y D L Y N G F V Q
atctacgctttacgcaacggagaagtaaaagttgaaatccaaggtgccttctatacgatc 4860
1620 I Y A L R N G E V K V E I Q G A F Y T I
tacgatggaaaacgtatcaaagtaacatcaaccggcggtaaattgcgcgattccaataga 4920
1640 Y **D G K R** I K V T S T G G K L R D S N R
ggattgtgcggtaaattcagtaacgacaaatacgaagacttcaccgttcctgctgactgc 4980
1660 **G L C G** K F S N D K Y E D F T V P A D C
gttgtatctgatccacgcaaattcaccgacagttaccaagtggaaaagagcaagagacca 5040
1680 V V S D P R K F T D S Y Q V E K S K R P
caacgagacagtcaagaatgcgtcgccaaagtaatgcccttgtatgctagagtcggattc 5100
1700 Q R D S Q E C V A K V M P L Y A R V G F
cgaaaatccggtgaggcaagaatgaatctgagaactcgctatgccgaacaaaacggcgaa 5160

```
1720   R  K  S  G  E  A  R  M  N  L  R  T  R  Y  A  E  Q  N  G  E
       atctgtttctcccttcaacccctacccacttgcaaaggaagtccaagaagaaccgagagt    5220
1740   I  C  F  S  L  Q  P  L  P  T  C  K  G  S  P  R  R  T  E  S
       gaaccggtcgaagctcactgcatccagaaaactaagtctgctctgtatttcaaggctcaa    5280
1760   E  P  V  E  A  H  C  I  Q  K  T  K  S  A  L  Y  F  K  A  Q
       atcgaccaaggtgccaacccagatttcagccagaagagcctaaccaggcatgtcgatatg    5340
1780   I  D  Q  G  A  N  P  D  F  S  Q  K  S  L  T  R  H  V  D  M
       aaagttcacaaacaatgtaattaaaattctataatgaccttatgtattattatttagtta    5400
1800   K  V  H  K  Q  C  N  *
       agaattctgtaaaaaatgcataaaatttaaattaaaatatctcggtgtctgttcaggcag    5460
       ataactaatcgggggaatacttttcatgcaaccagattaaaaaa                    5504
```

Figure S1. Nucleotide and amino acid sequence of the RfVg cDNA. Nucleotides sequence is numbered on the right side and the deduced amino acid sequence on the left side of each line. The DGXR and GL/ICG conserved motifs are present at C-terminal and indicated with underline. The five possible cleavage site (RXXR) motifs are boxed. The signal peptide is shown by a double underline. The glycosylation sites (NXS/T) are indicated with a bold underline, whereas the phosphorylated tyrosine (Y), threonine (T), and serine (S) residues are indicated with light dark shaded frames. The C-terminal cysteine residues are red circled.

Table S1. List of primers used for RACE PCR, RT-PCR, and RNAi experiments to amplify the *RfVg* gene transcript.

Primers	Sequences
**RfVgF1**	5′ CAAGAGACCACAACGAGACAGTCAA 3′
**RfVgRTF1**	5′ TCTGGGGAGTAGCTCTAGCTTCGAT 3′
**RfVgRTR1**	5′ CTGCCTACGTTTTGTTCAGAGATCC 3′
**Adopter primer 1 (AP1)**	5′ CCATCCTAATACGACTCACTATAGGGC 3′
**RfVgRNAiF1**	5′ TAATACGACTCACTATAGGGGATTTCCTCAGCTACAGCCAGTTGA 3′
**RfVgRNAiR1**	5' TAATACGACTCACTATAGGGAGTTCACCTTGCCTGATACTTGAGC 3'
**RfVgRTF2**	5′ CTGCCTACGTTTTGTTCAGAGATCC 3′
**RfVgRTR2**	5' GACAACTTACCTCAAGCCATTCGTG 3'
**TubulinRfer-F**	5′ GCTACCTTCATCGGCAACTC 3′
**TubulinRfer-R**	5' CGGTGGCTTCTTGGTATTGT 3'

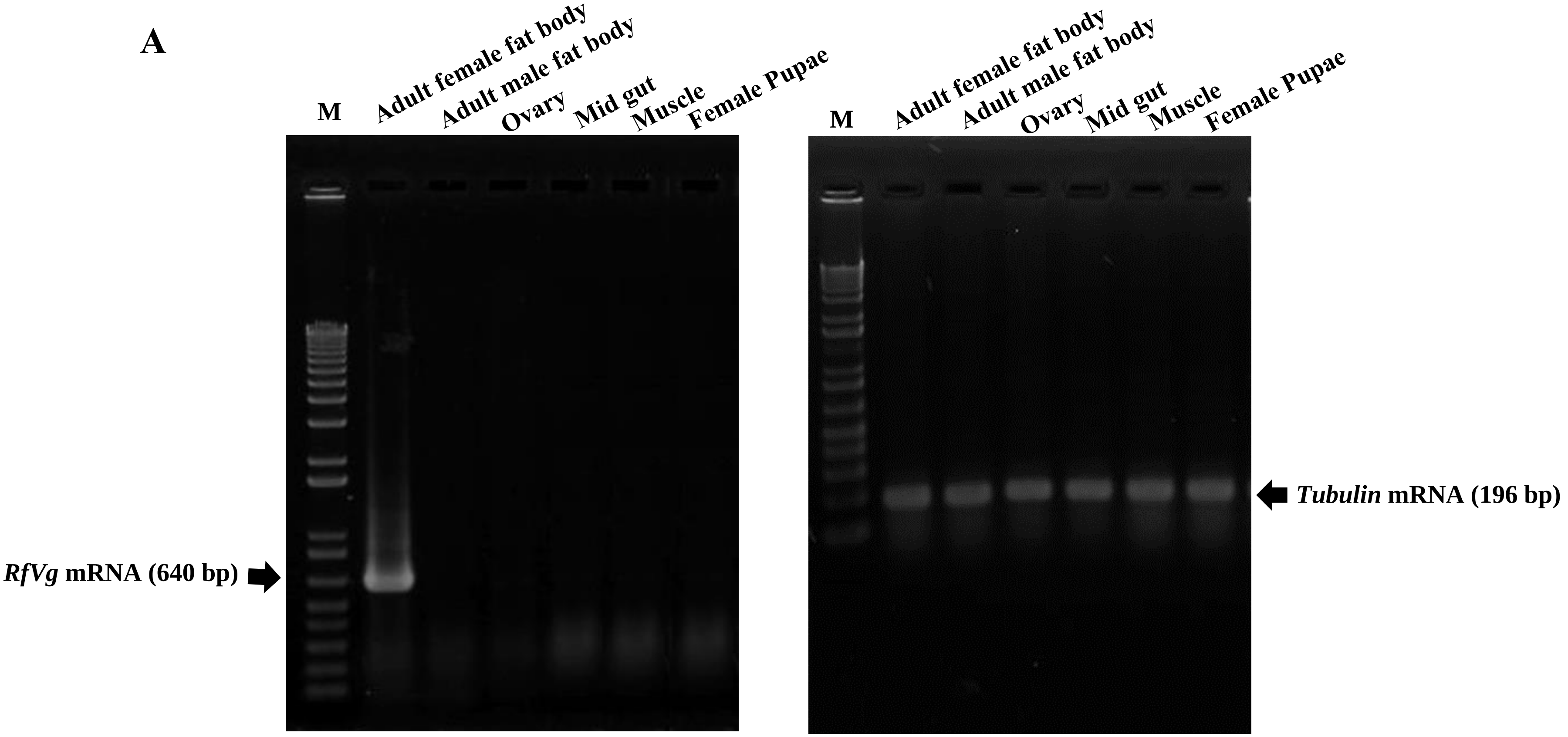

Figure 2. Expression pattern and temporal traits of *RfVg* gene transcription. Expression pattern of *RfVg* and *tubulin* genes from different tissues of *Rhynchophorus ferrugineus* was analyzed by RT-PCR. (Uncropped Gel)

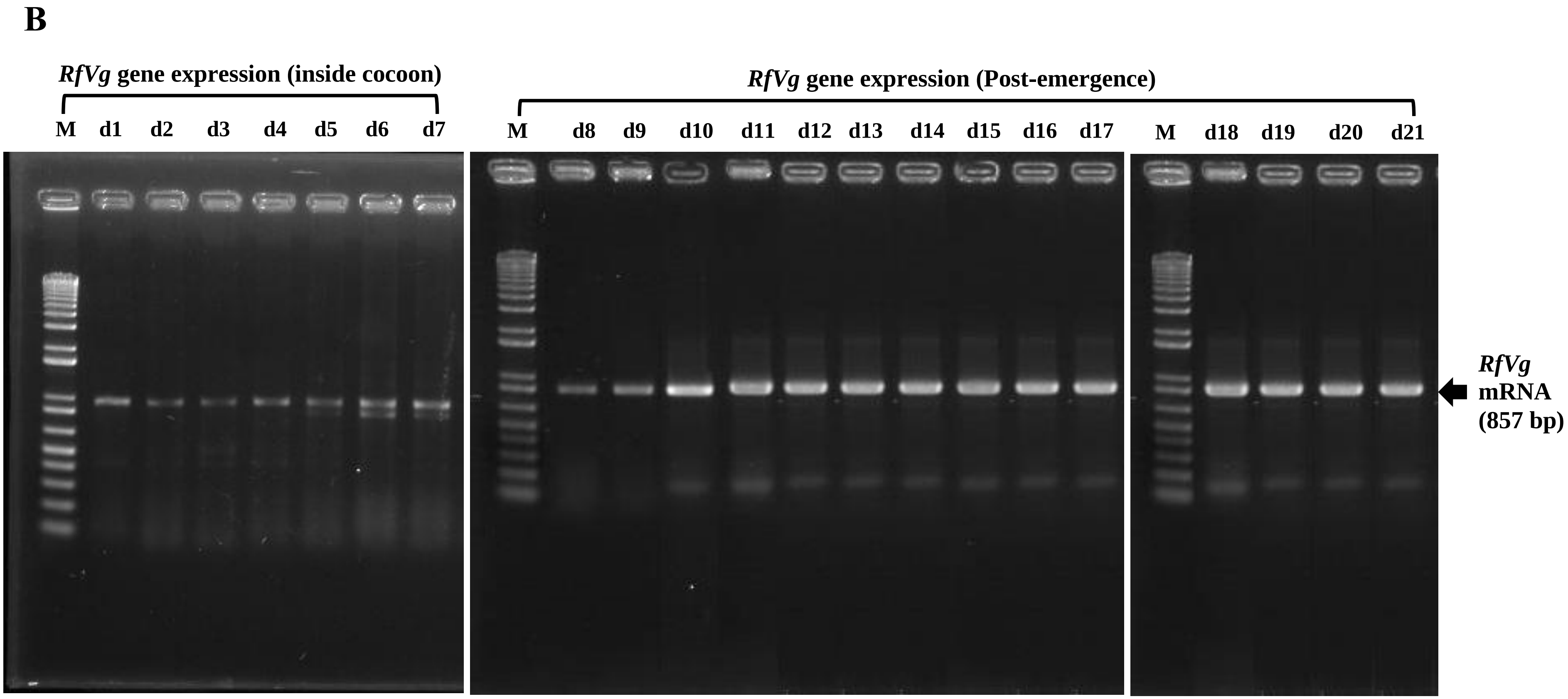

Figure 2. Expression pattern and temporal traits of *RfVg* gene transcription. The *RfVg* genes expression profile up to three weeks in the adult *R. ferrugineus* females analyzed by RT-PCR. (Uncropped Gel)

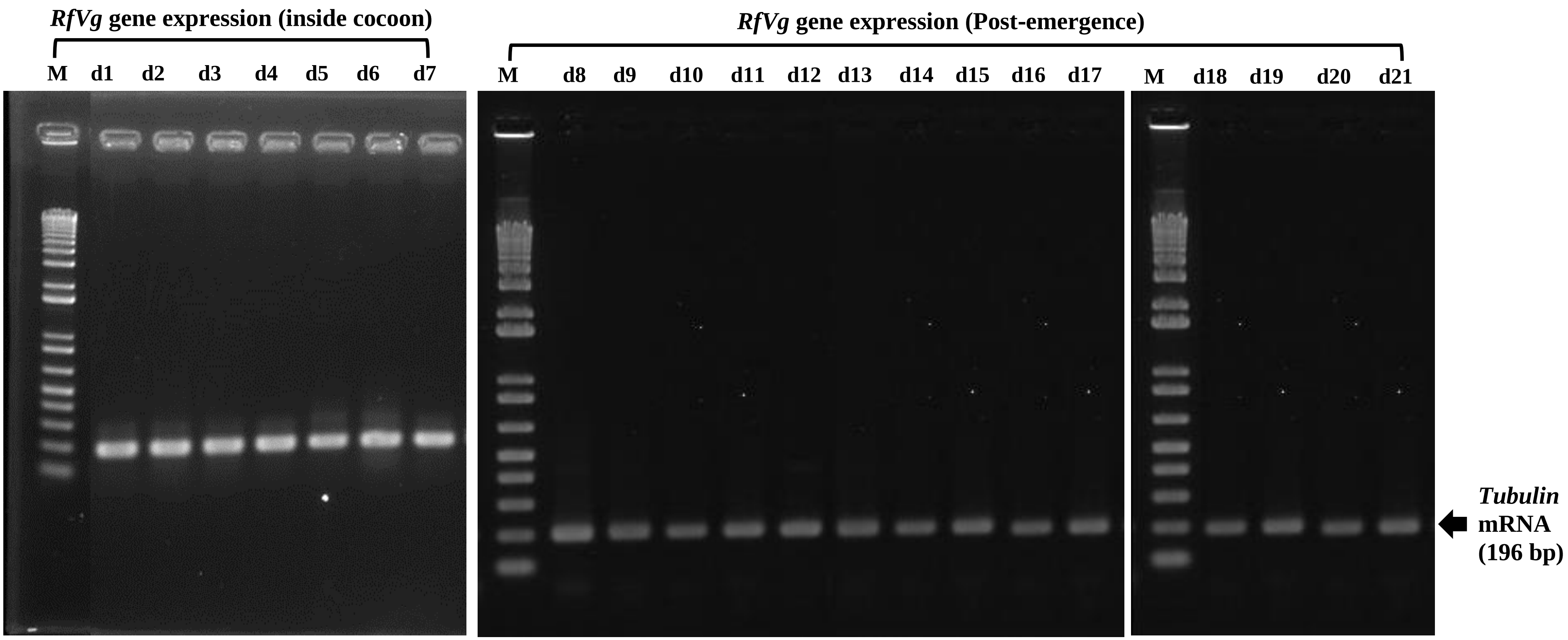

Figure 2. The *tubulin* genes expression profile up to three weeks in the adult *R. ferrugineus* females analyzed by RT-PCR. (Uncropped Gel)

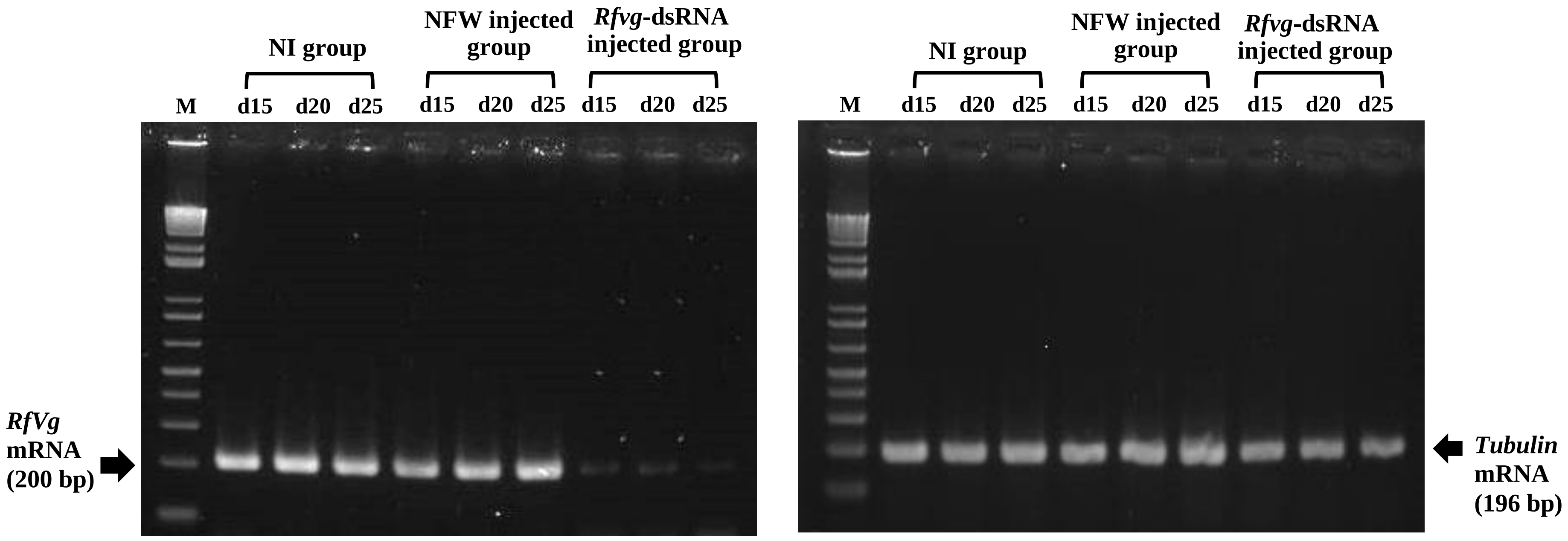

Figure-4. Validation of RNAi and relative expression analysis of *RfVg* gene through RT-PCR. (Uncropped Gel)

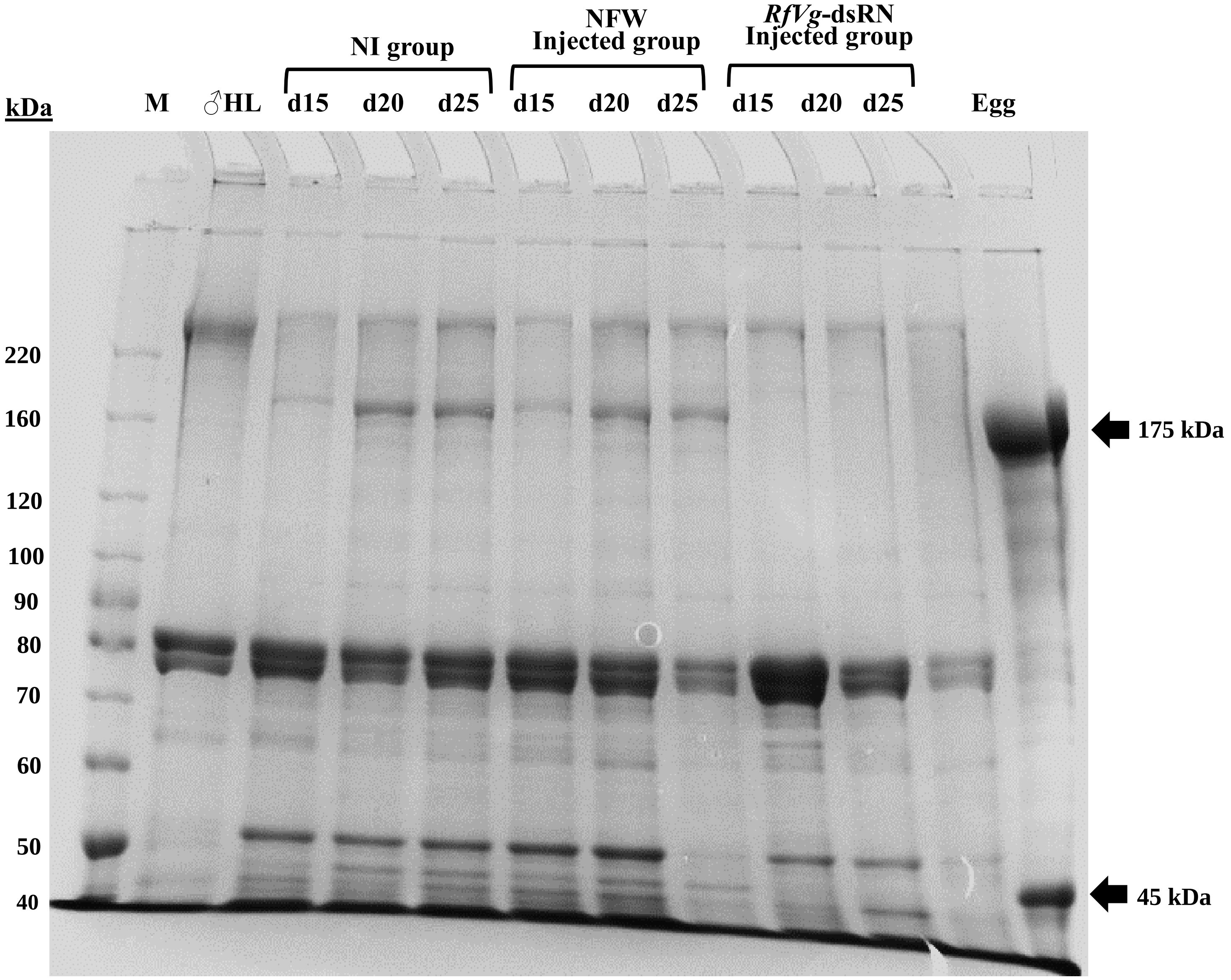

Figure 5. Validation of RNAi and expression analysis of *RfVgs* by SDS-PAGE. (Uncropped Gel)
